# Spectrophotometric Evaluation of the Masking Ability of 3D-Printed Ceramic-Filled Hybrid Veneers on Discolored Dental Substrates

**DOI:** 10.3390/dj14060349

**Published:** 2026-06-08

**Authors:** Alexandra Cristina Măroiu, Anca-Elena Anghel-Lorinți, Marius Leretter, Raul Rotar, Adrian Cândea, Anca Jivănescu

**Affiliations:** 1 Department of Prosthodontics, University of Medicine and Pharmacy “Victor Babes”, B-dul Revolutiei 1989, No. 9, 300580 Timisoara, Romania; maroiu.alexandra@umft.ro (A.C.M.); rotar.raul@umft.ro (R.R.); candea.adrian@umft.ro (A.C.); jivanescu.anca@umft.ro (A.J.); 2 TADERP Research Center, Department of Prosthodontics, University of Medicine and Pharmacy “Victor Babes”, B-dul Revolutiei 1989, No. 9, 300580 Timisoara, Romania

**Keywords:** 3D printing, dental veneers, tooth discoloration, spectrophotometry, ceramic-filled hybrid material

## Abstract

**Background/Objectives**: Tooth discoloration can influence the esthetic outcome of restorative treatments. Recently, 3D-printed ceramic-filled hybrid materials have been introduced for dental restorations using digital workflows. The aim of this in vitro study was to evaluate the influence of 3D-printed ceramic-filled hybrid veneers on the final color of discolored teeth using spectrophotometric measurements. **Methods**: Twenty-five extracted human anterior teeth without caries or restorations were prepared for veneer restorations using standardized reduction protocols. Artificial discoloration was induced by applying light-cured color coatings to the buccal surfaces of the specimens. The prepared teeth were digitally scanned, and veneers with a thickness of 1 mm were designed and fabricated using a 3D printing system and a ceramic-filled hybrid material. Color measurements were performed with a spectrophotometer and recorded in the Commission Internationale de l’Éclairage L*a*b* (CIELAB) color system. Measurements were obtained at four stages: after creation of discoloration, after two weeks of immersion in physiological saline solution, after veneer placement using neutral try-in gel, and after two months of immersion following veneer placement. Color differences were calculated using three color difference formulas (ΔE*ab, ΔE94, and ΔE00). **Results**: The placement of the 3D-printed veneers produced substantial modifications in the optical characteristics of the discolored substrates, reflected by reduced chroma values and significant color differences between the baseline and veneer stages. After two months of immersion, only minor variations in color coordinates were observed. The calculated color differences between the veneer stage and the post-immersion stage remained low across all evaluated color difference formulas, indicating good short-term color stability of the veneered specimens. **Conclusions**: Within the limitations of this pilot in vitro study, 3D-printed ceramic-filled hybrid veneers demonstrated the ability to effectively modify the color of discolored substrates while maintaining relatively stable optical properties after two months of immersion. These restorations may represent a promising and cost-effective option for the esthetic management of discolored teeth.

## 1. Introduction

Dental esthetics plays an important role in contemporary restorative dentistry, and tooth color is one of the most relevant parameters influencing the appearance of the dentition. Alterations in tooth color may occur because of intrinsic or extrinsic factors, including developmental defects, aging, trauma, restorative materials, or staining from dietary and environmental sources. Such changes may lead to patient dissatisfaction and frequently require restorative or prosthetic intervention to restore a more uniform dental appearance [1,2,3].

Different clinical approaches have been proposed for the management of tooth discoloration, including bleaching procedures, direct composite restorations, and indirect restorations such as ceramic veneers or crowns. Among these options, veneers represent a conservative restorative solution that allows modification of tooth color, shape, and surface morphology while preserving a substantial amount of dental tissue [4,5]. Conventional ceramic veneers have demonstrated favorable optical properties and long-term clinical performance; however, their fabrication usually requires laboratory procedures and can involve relatively high costs and complex manufacturing processes [6,7].

Recent developments in digital dentistry have introduced additive manufacturing technologies as potential alternatives for the fabrication of dental restorations. Three-dimensional (3D) printing has gained increasing attention due to its ability to produce restorations through fully digital workflows, potentially reducing manufacturing time and simplifying laboratory procedures. In addition, new printable dental materials have been developed, including ceramic-filled hybrid resins designed to combine the mechanical properties of polymer-based materials with the optical characteristics of ceramic fillers [8,9,10]. These materials have been proposed for the fabrication of temporary or definitive restorations, including crowns, bridges, and veneers.

Despite the increasing use of 3D printing technologies in dentistry, limited information is available regarding the optical behavior of these materials when used as thin restorations intended to mask underlying tooth discoloration. The final color of veneer restorations is influenced by several factors, including the color of the underlying substrate, the thickness of the restorative material, and the optical properties of the material itself. Spectrophotometric evaluation using the CIELAB color system (L*, a*, b*) represents a widely used method for the objective assessment of color differences in dental research [11,12,13].

Therefore, the aim of the present in vitro study was to evaluate the influence of 3D-printed ceramic-filled hybrid veneers on the final color of discolored teeth using spectrophotometric measurements. The color parameters of the specimens were analyzed at different experimental stages to assess the masking effect of the printed veneers and the stability of the resulting color over time. The hypothesis of the present study was that the placement of 3D-printed ceramic-filled hybrid veneers would not produce clinically significant color modifications in discolored dental substrates and would not result in measurable changes in color stability over time.

## 2. Materials and Methods

### 2.1. Sample Selection

This in vitro experimental study included twenty-five extracted human anterior teeth selected for the experimental protocol. The teeth had been extracted for periodontal reasons and were included only if they presented intact vestibular surfaces, with no carious lesions, restorations, cracks, or structural defects that could influence color assessment or veneer adaptation. All specimens were carefully inspected prior to inclusion in the study using visual examination under magnification to detect cracks, restorations, structural defects, or surface irregularities that could influence color measurements or veneer adaptation. Teeth presenting any visible defects were excluded from the experimental protocol.

After extraction, the specimens were cleaned of residual soft tissues and stored in 0.9% physiological saline in order to simulate in vivo conditions and to prevent dehydration throughout the experimental procedures.

Each tooth represented an independent experimental unit in the study. Spectrophotometric measurements were performed for each specimen at the predefined experimental stages in order to evaluate the color modifications produced by the 3D-printed veneers and the stability of the resulting color over time.

The study was conducted in accordance with the Declaration of Helsinki and it was approved by the Ethical Committee of University of Medicine and Pharmacy “Victor Babes” of Timisoara, Romania.

### 2.2. Tooth Preparation

All tooth preparations were performed by the same operator in order to standardize the preparation protocol and minimize operator-related variability. The preparations were carried out under 4× magnification using a dental magnification system (Univet, Rezzato, Italy).

The vestibular surface of each specimen was reduced by approximately 0.5 mm, following the natural two-dimensional planes of the facial surface. The incisal edge was reduced by 1 mm using a butt-joint finishing design in order to allow uniform veneer thickness. A chamfer finishing line with an approximate depth of 0.5 mm was created at the level of the gingival margin.

Tooth preparation was performed using a standardized set of diamond burs (Komet Dental, Lemgo, Germany), including depth-cutting burs 0.20 mm diameter for initial reduction, cylindrical coarse-grained burs 0.10 mm diameter for shaping, and fine-grained finishing burs 0.10 mm diameter for surface refinement. Final surface smoothing was performed using polishing instruments in order to obtain a uniform preparation surface.

**Figure 1:**
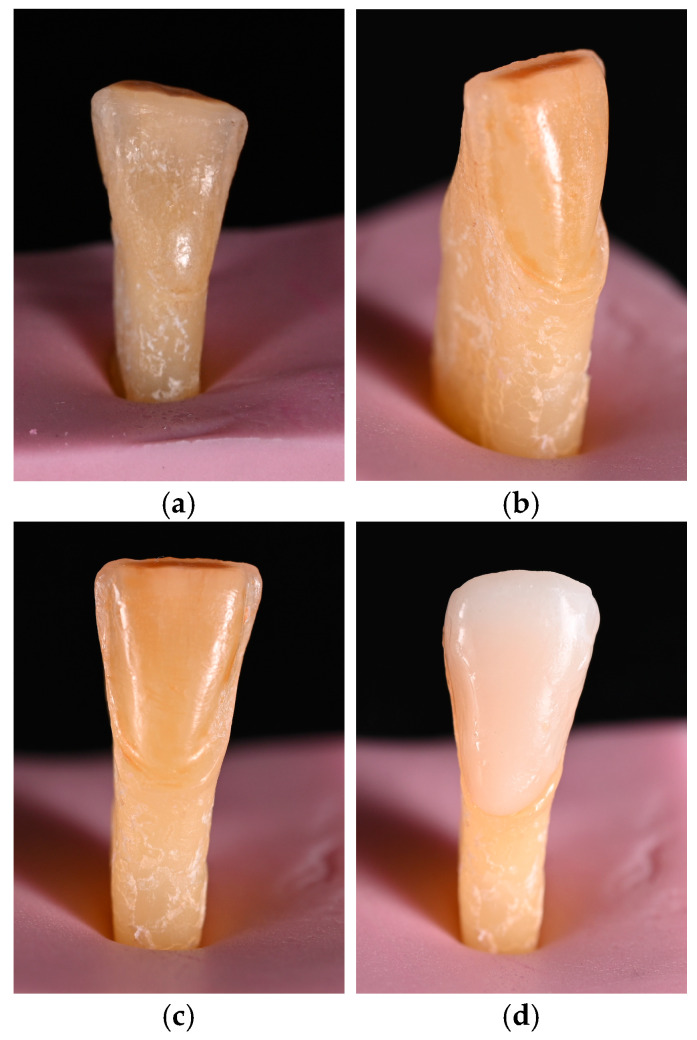
Standardized tooth preparation protocol for veneer restorations. (**a**) Initial tooth morphology; (**b**,**c**) prepared tooth from different perspectives, (**d**) 3D-printed veneer positioned on the prepared substrate.

The standardized preparation protocol used in this study is illustrated in Figure 1, which shows the sequential stages of tooth preparation, including the initial morphology, vestibular reduction, incisal reduction, and final preparation design.

The main preparation parameters used for veneer fabrication are summarized in Table 1.

### 2.3. Creation of Artificial Discoloration

In order to simulate different degrees of tooth discoloration, light-cured color characterization materials (Optiglaze Color, GC Corporation, Tokyo, Japan) were applied to the buccal surfaces of the prepared teeth. These materials allow controlled modification of the optical properties of the substrate and are commonly used for the characterization of dental restorative materials.

**Figure 2:**
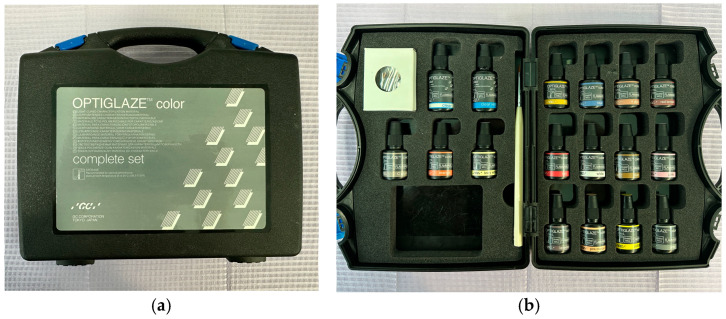
Light-cured color characterization materials used for the creation of artificial tooth discoloration (Optiglaze Color, GC Corporation, Tokyo, Japan). (**a**) Optiglaze Color complete kit; (**b**) individual color bottles used for the experimental characterization.

The Optiglaze Color system used in this study is presented in Figure 2, which shows the complete material kit and the individual color bottles employed to create artificial chromatic variations between the experimental samples.

Different shades from the Optiglaze Color system were selected to induce visible discoloration of the dental substrates. The tones included grey, orange, red-brown, and clear shades, which were applied in thin layers on the vestibular surfaces of the prepared teeth using a fine brush. The characterization materials were applied in thin and uniform layers using a controlled clinical application technique in order to standardize the thickness of the discoloration layer across all specimens. Polymerization was performed using the same calibrated light-curing unit under identical exposure conditions to ensure consistent optical properties among samples.

After application, the materials were polymerized using a dental light-curing unit according to the manufacturer’s instructions. The artificially induced discoloration served as the baseline condition for the subsequent evaluation of the masking effect produced by the printed veneers. The samples were stored in 0.9% physiological saline solution within an incubator at 37 °C (Cultura, Ivoclar Vivadent AG, Schaan, Liechtenstein) for two weeks.

### 2.4. Digital Scanning and Veneer Fabrication

The prepared teeth were digitized using an intraoral scanner (Medit i700, Medit Corp., Seoul, Republic of Korea). The scanning procedure allowed the acquisition of digital models of the prepared specimens, which were subsequently used for the computer-aided design of veneer restorations.

Veneers with a standardized thickness of approximately 1 mm were designed using CAD software and fabricated through a stereolithography (SLA) 3D printing workflow. The printing process was performed using a resin-based 3D printer (Prusa SL1, Prusa Research, Prague, Czech Republic), employing a ceramic-filled hybrid resin material (SprintRay Ceramic Crown, SprintRay Inc., Los Angeles, CA, USA) in shade A1. Following the printing procedure, the restorations were subjected to post-processing, including washing and photopolymerization, using a dedicated curing and washing unit (Prusa CW1, Prusa Research, Prague, Czech Republic), according to the manufacturer’s recommendations. This step ensured complete polymerization of the material and optimal mechanical and optical properties. After post-processing, the restorations were inspected and prepared for placement. The fabricated veneers were positioned on the prepared substrates using a neutral try-in paste (Variolink Esthetic Try-In, Ivoclar Vivadent, Schaan, Liechtenstein) in order to reproduce the optical conditions of the final luting procedure during color evaluation.

The veneers were seated using a standardized clinical positioning procedure, and excess try-in material was carefully removed prior to color measurement. This protocol ensured consistent seating conditions and minimized variability in the thickness of the intermediate layer.

**Figure 3:**
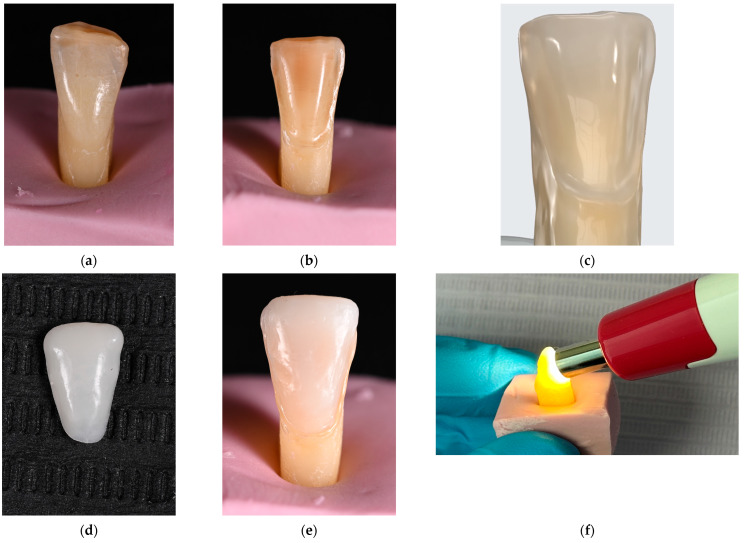
Experimental workflow of the study. (**a**) extracted anterior tooth before preparation; (**b**) prepared tooth for veneer restoration; (**c**) digital model obtained after scanning of the prepared specimen; (**d**) 3D-printed ceramic-filled hybrid veneer; (**e**) veneer positioned on the prepared tooth using try-in paste; (**f**) spectrophotometric color measurement.

The main stages of the experimental workflow, including digital acquisition, additive manufacturing, post-processing, and clinical simulation, are illustrated in Figure 3.

### 2.5. Spectrophotometric Color Measurement

Color measurements were performed using a dental spectrophotometer (VITA Easyshade V—the last generation, VITA Zahnfabrik, Bad Säckingen, Germany). The measurement procedure used in this study is illustrated in Figure 3f, where the spectrophotometer probe is positioned on the buccal surface of the specimen during color evaluation. After each measurement, the spectrophotometer was calibrated in accordance with manufacturer’s instructions. The color of each specimen was recorded according to the CIELAB color system, which includes the parameters L* (lightness), a* (red–green coordinate), and b* (yellow–blue coordinate).

Measurements were obtained from the central area of the buccal surface of each specimen for five consecutive readings, and the mean value was used for analysis in order to standardize the evaluation conditions. No positioning jig or custom matrix was used during the measurements, as the limited size and clearly defined geometry of the buccal surface allowed consistent probe positioning. Repeated measurements were performed in the same central region to ensure reproducibility and minimize measurement variability.

Color measurements were performed at four different experimental stages:

1. After the creation of artificial discoloration;
2. After two weeks of immersion in physiological saline solution;
3. After veneer placement using a neutral try-in paste (Variolink Esthetic Try-In, Ivoclar Vivadent);
4. After two months of immersion in physiological saline solution.

The measurements obtained at these stages allowed the evaluation of the color changes produced by the printed veneers and the stability of the resulting color over time.

### 2.6. Statistical Analysis

The spectrophotometric data was organized according to the four experimental stages: baseline after artificial discoloration (T0), after two weeks of immersion in physiological saline solution (T1), after veneer placement using neutral try-in paste (T2), and after two months of immersion (T3). For each specimen and each experimental stage, five consecutive spectrophotometric measurements were recorded in the center of the buccal surface, and the mean value was used for further analysis in order to reduce instrument-related variability.

Descriptive statistics were calculated for the CIELAB color coordinates L*, a*, and b*, and the results were expressed as mean ± standard deviation (SD) for each experimental stage.

Color differences between selected experimental stages were calculated using three commonly reported color difference formulas in dental color science: the classical CIELAB color difference (ΔE*ab), the CIE94 color difference (ΔE94), and the CIEDE2000 color difference (ΔE00). The use of multiple color difference metrics allowed verification of the consistency of the observed color changes across different calculation methods and improved the methodological robustness of the analysis.

The classical CIELAB color difference was calculated using the following equation:

$$\Delta E*ab=\sqrt{\left(L_{2}-L_{1}{)}^{2}+(a_{2}-a_{1}{)}^{2}+(b_{2}-b_{1}{)}^{2}\right.}$$

The CIE94 color difference was calculated according to the following equation:

$$\Delta E_{94}=\sqrt{{\left(\frac{∆L}{k_{L}S_{L}}\right)}^{2}+{\left(\frac{∆C}{k_{C}S_{C}}\right)}^{2}+{\left(\frac{∆H}{k_{H}S_{H}}\right)}^{2}}$$

where ΔL, ΔC, and ΔH represent the differences in lightness, chroma, and hue between two measurements, respectively, and $k_{L}$, $k_{C}$, and $k_{H}$ are parametric correction factors set to 1 under reference conditions.

The CIEDE2000 color difference was calculated according to the following equation:

$$\Delta E_{00}=\sqrt{{\left(\frac{\Delta L^{\prime }}{k_{L}S_{L}}\right)}^{2}+{\left(\frac{\Delta C^{\prime }}{k_{C}S_{C}}\right)}^{2}+{\left(\frac{\Delta H^{\prime }}{k_{H}S_{H}}\right)}^{2}+R_{T}\left(\frac{\Delta C^{\prime }}{k_{C}S_{C}}\right)\left(\frac{\Delta H^{\prime }}{k_{H}S_{H}}\right)}$$

where ΔL′, ΔC′, and ΔH′ represent the corrected differences in lightness, chroma, and hue, and RT is a rotation term accounting for the interaction between chroma and hue differences.

Two principal color difference comparisons were considered: T0–T2, in order to evaluate the masking effect of the 3D-printed veneers on the discolored substrates, and T2–T3, in order to assess the stability of the final color after immersion.

Because of the limited sample size and repeated-measures design, non-parametric statistical methods were used. Overall differences in the color coordinates across the four experimental stages were assessed using the Friedman test. Pairwise comparisons of the main experimental stages were performed using the Wilcoxon signed-rank test. Effect sizes were calculated for the pairwise comparisons in order to support the interpretation of the observed differences. Statistical significance was set at *p* < 0.05.

A priori sample size estimation was performed using MedCalc Statistical Software Version 23.4.0—64 bit, licensed to Universitatea Victor Babes Timisoara (MedCalc Software Ltd., Ostend, Belgium), based on a significance level of 0.05 and a statistical power of 80%. The analysis indicated that the inclusion of twenty-five independent specimens was sufficient to detect clinically relevant differences in color parameters across the experimental stages. For each specimen, five consecutive spectrophotometric measurements were performed, and the mean value was used for statistical analysis in order to reduce measurement variability.

Statistical analysis was performed using MedCalc Statistical Software (MedCalc Software Ltd., Ostend, Belgium).

## 3. Results

### 3.1. Spectrophotometric Measurements of the Discolored Substrates

The spectrophotometric measurements recorded after the creation of artificial discoloration are presented in Table 2. The initial color coordinates showed variability between samples, reflecting the different chromatic alterations induced on the prepared substrates using the characterization materials.

The measured L*, a*, and b* values indicated differences in both lightness and chromatic components among the specimens, confirming that the artificial discoloration procedure produced heterogeneous substrate colors suitable for evaluating the masking ability of the printed veneers.

After two weeks of immersion in physiological saline solution within an incubator at 37 °C, additional spectrophotometric measurements were performed. The values obtained at this stage are presented in Table 3. Compared with the baseline measurements, slight variations in the CIELAB coordinates were observed, mainly affecting the chromatic components rather than the overall lightness of the specimens.

These measurements established the baseline optical characteristics of the discolored substrates and served as reference values for the subsequent evaluation of color modifications produced by the placement of the 3D-printed veneers.

### 3.2. Color Parameters After Veneer Placement

The spectrophotometric measurements recorded after the placement of the 3D-printed veneers using neutral try-in paste are presented in Table 4.

Placement of the veneers produced clear modifications in the measured CIELAB color coordinates, indicating the optical influence of the printed restorations on the underlying discolored substrates.

In general, the placement of the veneers was associated with a reduction in chromatic intensity of the underlying substrates, as reflected by lower b* values in most specimens. Variations in the L* coordinate were also observed, indicating changes in lightness after veneer placement.

These measurements represent the color parameters of the tooth–veneer assemblies immediately after veneer placement (T2) and served as reference values for the subsequent evaluation of color stability following the aging period.

### 3.3. Color Stability After Immersion

The spectrophotometric measurements obtained after two months of immersion in physiological saline solution within an incubator at 37 °C are presented in Table 5. The recorded CIELAB color coordinates showed only minor variations compared with the measurements obtained immediately after veneer placement.

In most specimens, slight changes in the L*, a*, and b* coordinates were observed, indicating minor modifications in lightness and chromatic components during the immersion period. However, the overall color parameters of the tooth–veneer assemblies remained relatively stable throughout the observation interval.

The magnitude of these variations remained limited, suggesting a relatively stable optical behavior of the tooth–veneer assemblies during the immersion period.

These findings indicate that the final color obtained after veneer placement remained largely stable after the second immersion period (T3), performed under controlled laboratory conditions.

### 3.4. Color Difference (ΔE) and Statistical Analysis

**Figure 4:**
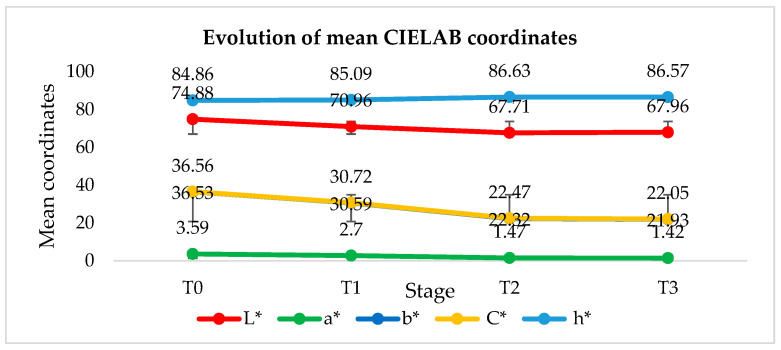
Evolution of the mean CIELAB color coordinates (L*, a*, and b*) during the experimental stages (T0–T3). Error bars represent standard deviation.

Descriptive statistics for the CIELAB color coordinates (L*, a*, and b*) recorded at the four experimental stages are summarized in Table 6. The mean values indicated progressive variations in the optical characteristics of the specimens throughout the experimental protocol. The evolution of the mean CIELAB coordinates during the experimental stages is illustrated in Figure 4.

The mean b value decreased from 36.53 ± 5.87 at T0 to 22.32 ± 5.41 after veneer placement (T2), indicating a marked reduction in the yellow chromatic component after the application of the printed veneers. Slight variations were also observed in the a* coordinate, with lower mean values recorded after veneer placement. A corresponding reduction in chroma (C*) values was also observed after veneer placement, indicating decreased color saturation, while the hue angle (h*) remained relatively stable across the experimental stages.

Color differences between selected experimental stages were calculated using three color difference formulas (ΔE*ab, ΔE94, and ΔE00), as described in the Statistical Analysis section. Two principal comparisons were considered: baseline discoloration versus veneer placement (T0–T2), in order to evaluate the masking effect of the 3D-printed veneers, and veneer placement versus the two-month immersion stage (T2–T3), in order to assess the stability of the final color.

**Figure 5:**
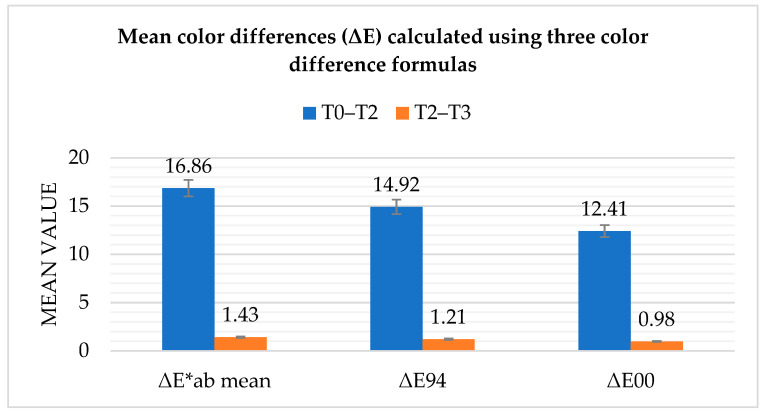
Mean color differences (ΔE*ab, ΔE94, and ΔE00) calculated between baseline discoloration and veneer placement (T0–T2) and between veneer placement and two-month immersion (T2–T3). Error bars represent standard deviation.

Mean color differences (ΔE*ab, ΔE94, and ΔE00) calculated between selected experimental stages are presented in Table 7 and illustrated in Figure 5.

The mean color difference between the discoloration stage and the veneer stage (ΔE*ab T0–T2) was 16.86 ± 4.35, indicating substantial color modification after veneer placement. Comparable values were obtained using the CIE94 (14.92 ± 3.88) and CIEDE2000 (12.41 ± 3.21) formulas, confirming the consistency of the observed color changes across different calculation methods. In contrast, the color differences recorded between the veneer stage and the two-month immersion stage (ΔE*ab T2–T3) were considerably lower, with a mean value of 1.43 ± 0.79. Similar low values were obtained using the CIE94 (1.21 ± 0.66) and CIEDE2000 (0.98 ± 0.54) formulas, indicating good short-term color stability of the veneered specimens.

The mean color differences calculated between the main experimental stages are presented in Figure 5.

Because of the limited sample size and the repeated-measures design of the study, non-parametric statistical tests were applied. Overall differences among the four experimental stages were evaluated using the Friedman test (Table 8), which indicated statistically significant differences in all evaluated color coordinates (L*, a*, and b*) across the experimental stages (*p* < 0.001).

Pairwise comparisons were subsequently performed using the Wilcoxon signed-rank test (Table 9). The comparison between the discoloration stage and the stabilization stage (T0–T1) demonstrated statistically significant differences in most evaluated parameters, reflecting the initial color changes occurring during the immersion period. A further statistically significant reduction in chromatic values was observed between the stabilization stage and the veneer placement stage (T1–T2), confirming the substantial optical modification produced by the placement of the 3D-printed veneers.

In contrast, the comparison between the veneer placement stage and the two-month immersion stage (T2–T3) revealed no statistically significant differences for any of the evaluated color coordinates (*p* > 0.05). This finding indicates that the optical characteristics obtained immediately after veneer placement remained stable during the subsequent immersion period.

The Friedman test revealed statistically significant differences among the experimental stages for all evaluated color coordinates (*p* < 0.001).

The observed statistical pattern therefore reflects two distinct phases of color change within the experimental protocol. The first phase corresponds to the pronounced color modification associated with veneer placement, as evidenced by the significant differences detected between the pre-restorative and post-restorative stages. The second phase corresponds to the stabilization of the optical properties of the tooth–veneer assemblies, as indicated by the absence of significant differences between the veneer stage and the post-immersion stage.

Specimens with darker baseline shades demonstrated significantly higher ΔE values compared to lighter shades, indicating increased masking difficulty in these conditions. This finding confirms that substrate shade is a critical factor influencing the masking performance of ceramic veneers.

These findings confirm that the placement of the 3D-printed veneers produced a significant and clinically relevant modification of the color coordinates of the discolored substrates, while the subsequent aging period produced only minimal variations in the measured parameters.

## 4. Discussion

The present study investigated the influence of 3D-printed ceramic-filled hybrid veneers on the color appearance of discolored dental substrates using spectrophotometric measurements. The results demonstrated consistent improvements in the color characteristics of the discolored substrates following veneer placement, indicating an effective modification of their optical appearance. In relation to the study hypothesis, the findings showed that the placement of the tested veneers produced measurable and clinically relevant changes in color parameters, particularly through an increase in lightness and a reduction in chromatic intensity. In contrast, the color changes observed after the two-month immersion period were minimal, suggesting relatively stable short-term optical behavior under the experimental conditions. These findings support the ability of the tested material to improve the esthetic appearance of discolored substrates while maintaining acceptable short-term color stability.

A key finding of this study was the significant reduction in the b* coordinate following veneer placement, reflecting a decrease in the yellow chromatic component of the discolored substrates. This observation is consistent with the expected masking behavior of restorative materials with reduced translucency and adequate thickness. The masking ability of veneer restorations is influenced by several factors, including restoration thickness, material optical properties, and the color of the underlying dental substrate [14,15,16].

Previous investigations have demonstrated that both thickness and material translucency are critical determinants of the final optical outcome of veneer restorations, particularly when masking discolored substrates [17,18,19]. Ceramic restorations with greater thickness generally exhibit improved masking ability, particularly when underlying discoloration is present [20]. In the present study, veneers with an approximate thickness of 1 mm were used, which represents a clinically relevant compromise between conservative preparation and sufficient masking ability.

The calculated color differences between the discoloration stage and the veneer stage showed a mean ΔE*ab value of 16.86 ± 4.35, indicating a pronounced color modification produced by the veneer restorations. Comparable values were obtained using the CIE94 (ΔE94 = 14.92 ± 3.88) and CIEDE2000 (ΔE00 = 12.41 ± 3.21) formulas, confirming the consistency of the observed color changes across different calculation methods. This magnitude of color difference clearly exceeds commonly reported perceptibility and acceptability thresholds in dentistry, confirming a substantial masking effect of the printed veneers.

In contrast, the color differences recorded after the two-month immersion period were considerably lower (mean ΔE*ab = 1.43 ± 0.79). Similarly low values were obtained using the CIE94 (ΔE94 = 1.21 ± 0.66) and CIEDE2000 (ΔE00 = 0.98 ± 0.54) formulas, indicating good short-term color stability of the veneered specimens. These values fall within commonly reported perceptibility thresholds, suggesting that the final color obtained after veneer placement remained relatively stable during the simulated aging period.

Importantly, the magnitude of the color differences observed in the present study reflects two distinct optical phenomena. The large color difference recorded between the discolored substrates and the veneered specimens (T0–T2) represents the masking capacity of the restorations, demonstrating their ability to effectively alter the initial discoloration. In contrast, the much smaller color difference recorded between the veneered stage and the post-immersion stage (T2–T3) represents the color stability of the veneered assemblies rather than an additional masking effect. This distinction is clinically relevant, as successful esthetic restorations must not only modify the initial color but also maintain stable optical properties over time.

To enhance the robustness and clinical relevance of the color analysis, three color difference formulas (ΔE*ab, ΔE94, and ΔE00) were calculated in the present study. While the traditional CIELAB formula (ΔE*ab) remains widely used in dental research, the CIE94 and CIEDE2000 formulas incorporate perceptual weighting factors that better reflect human color perception and are increasingly recommended for clinical color evaluation. The consistent trends observed across all three formulas in the present study support the reliability of the reported color changes.

Color differences in restorative dentistry are frequently interpreted using perceptibility and acceptability thresholds. Previous studies have suggested that ΔE*ab values of approximately 1–2 represent the threshold of perceptibility, while values above 3–5 may be considered clinically unacceptable under certain conditions [21,22,23]. Within this framework, the ΔE*ab values observed in the present study indicate that the veneers produced a clinically relevant modification of the initial discoloration while maintaining acceptable short-term color stability within commonly accepted perceptibility limits. The results highlight the importance of considering the initial tooth shade during treatment planning, as darker substrates may require increased restoration thickness or alternative material selection to achieve optimal esthetic outcomes.

Despite these promising findings, several methodological aspects must be carefully considered when interpreting the results. First, the discoloration model used in this study was based on the application of light-cured characterization materials on the tooth surface. While this approach allowed standardized and reproducible chromatic variation among specimens, it primarily simulates surface-level discoloration rather than intrinsic discoloration originating from dentin or internal tooth structure. In clinical scenarios, intrinsic discolorations—such as those caused by trauma, tetracycline staining, or fluorosis—may present different optical challenges, and the masking ability of veneer materials may therefore differ from the results observed in this study. Consequently, the present findings should be interpreted as representative of controlled experimental conditions rather than direct clinical performance.

Another important consideration is the use of a neutral try-in paste instead of a definitive resin-based luting cement during veneer placement. The optical properties of the luting agent, including its shade and refractive index, are known to influence the final color of veneer restorations. Try-in pastes are designed to approximate the optical behavior of resin cements; however, they do not perfectly replicate their polymerization-dependent optical changes. As a result, the final color outcome observed in this study may differ from that achieved under clinical conditions using definitive adhesive cementation. Future studies should incorporate different resin cement shades and polymerization protocols to provide a more comprehensive evaluation of the final optical outcome.

Color stability is a critical factor in the long-term success of esthetic restorations, particularly for resin-based and hybrid materials that may be susceptible to water sorption, matrix degradation, and surface alterations [24,25,26]. In the present study, the relatively low ΔE*ab, ΔE94, and ΔE00 values observed after two months of immersion suggest favorable short-term stability of the tested material. However, the aging protocol was limited to static immersion in physiological saline solution and did not incorporate additional clinically relevant factors such as thermal cycling, exposure to staining agents, or mechanical wear. These factors may significantly influence the long-term optical behavior of restorative materials. Therefore, the present results should be interpreted as indicative of short-term stability rather than long-term performance.

In addition to the intrinsic material properties, the use of an SLA-based additive manufacturing workflow introduces specific factors that may influence the optical behavior of the restorations. The layer-by-layer fabrication process and the post-curing protocol can affect the degree of monomer conversion and material homogeneity, both of which are directly related to translucency and color stability. The standardized post-processing performed using a dedicated curing unit may therefore have contributed to the favorable optical stability observed in the present study.

Additive manufacturing technologies have recently introduced new materials for dental restorations, including ceramic-filled hybrid resins designed for fully digital workflows [27,28,29]. The additive manufacturing workflow used in this study introduces additional variables that may influence optical outcomes, including printing orientation, layer thickness, and post-curing protocols. These parameters can affect the degree of monomer conversion, internal structure, and surface characteristics of the printed restorations, thereby influencing translucency and color stability. Compared with conventional subtractive ceramic fabrication, additive manufacturing may allow more efficient material use, simplified production processes, and increased accessibility of digital restorative workflows. These materials may offer practical advantages such as simplified fabrication procedures and reduced manufacturing costs compared with conventional ceramic restorations [30]. The present results therefore provide preliminary evidence supporting the optical performance of additively manufactured ceramic-filled hybrid materials in veneer-type restorations. The standardized post-processing protocol applied in this study may have contributed to the relatively stable optical behavior observed; however, variations in manufacturing parameters could lead to different outcomes. Further investigation of these factors is warranted to optimize the clinical performance of 3D-printed restorative materials. However, their optical behavior and long-term stability remain subjects of ongoing investigation.

Recent studies have further investigated the optical behavior and color stability of 3D-printed dental materials, highlighting the influence of printing orientation, post-curing protocols, and material composition on final optical outcomes [31,32,33,34,35,36,37]. These factors may affect polymerization efficiency, internal structure, and surface characteristics, ultimately influencing color stability and translucency. Continued investigation of these parameters is therefore essential for optimizing the clinical performance of additively manufactured restorative materials. Recent advances in digital dentistry have introduced software-based tools for smile design and color simulation that support the planning of esthetic restorations. Digital platforms such as 3Shape and Dynasmile allow clinicians to visualize the potential interaction between restoration thickness, material translucency, and the color of the underlying substrate before the fabrication of the final restoration. These technologies may improve the predictability of esthetic outcomes by enabling objective evaluation of restorative parameters and facilitating communication between clinicians, dental technicians, and patients. In addition, digital visualization tools may support more individualized treatment planning, particularly in cases involving discolored substrates where masking ability represents a critical clinical factor.

The present study has several limitations that should be acknowledged. First, the sample size was limited, and the investigation should therefore be considered a pilot study. Second, the experimental design was performed in vitro and does not fully replicate the complex conditions present in the oral environment. Factors such as salivary enzymes, dietary chromogens, thermal cycling, and mechanical wear were not reproduced in the present experimental model. Additionally, the observation period was limited to two months, and longer-term studies are required to evaluate the color stability of these materials under clinically relevant conditions.

Another limitation is the absence of direct comparison with conventional ceramic veneers or other CAD/CAM materials, which would allow a more comprehensive evaluation of the relative masking performance of 3D-printed restorations. Comparative investigations involving multiple restorative materials may provide a clearer understanding of the clinical advantages and limitations of additively manufactured hybrid restorations. These considerations are consistent with recent investigations reporting relatively stable optical properties of hybrid and CAD/CAM materials under simulated aging conditions [38,39,40].

One limitation of the present study is the use of try-in paste instead of definitive resin cement during color evaluation. Although try-in pastes are widely used to simulate the final color outcome of ceramic veneers, their optical properties may differ slightly from those of polymerized resin cement. Therefore, the results should be interpreted as predictive rather than definitive representations of clinical outcomes.

Despite these limitations, the present study provides important preliminary evidence regarding the optical performance of 3D-printed ceramic-filled hybrid veneers. The findings suggest that these materials are capable of producing clinically relevant color modifications in discolored substrates while maintaining acceptable short-term color stability. From a clinical perspective, the potential advantages of additive manufacturing, including simplified workflows, reduced material waste, and increased accessibility, make these materials a promising alternative to conventional restorative approaches. However, their adoption in routine clinical practice requires further validation through long-term and comparative studies.

Future research should focus on expanding the experimental design to include larger sample sizes, longer aging periods, and clinically relevant simulation protocols incorporating thermocycling, staining solutions, and mechanical loading. Additionally, comparative studies evaluating 3D-printed materials alongside conventional ceramic and CAD/CAM restorations would provide a more comprehensive understanding of their relative advantages and limitations. Such investigations are essential for establishing evidence-based guidelines for the clinical use of additively manufactured veneer restorations.

Recent investigations have increasingly focused on the optical performance and color stability of additively manufactured dental materials, reflecting the rapid evolution of digital restorative technologies. Several studies published in the past five years have reported that factors such as printing orientation, layer thickness, and post-curing protocols may significantly influence the optical properties and long-term color stability of 3D-printed resin-based restorations [41,42,43]. In particular, contemporary research has demonstrated that modern ceramic-filled hybrid and resin-based materials can achieve clinically acceptable color stability under simulated aging conditions, although variations in surface roughness and polymerization efficiency may still affect their optical behavior [44]. These findings support the growing clinical relevance of additive manufacturing workflows in esthetic dentistry and highlight the importance of standardized fabrication parameters for ensuring predictable esthetic outcomes in veneer-type restorations.

In addition, recent studies have emphasized the importance of objective color measurement techniques, particularly spectrophotometric analysis using advanced color difference formulas such as CIE94 and CIEDE2000, for evaluating the clinical acceptability of restorative materials. Contemporary evidence indicates that ΔE00 thresholds provide improved correlation with human visual perception and are increasingly recommended for clinical and research applications in esthetic dentistry [45]. The consistent use of standardized color evaluation methods therefore represents a key factor in improving the reproducibility and comparability of research findings related to masking ability and color stability of modern restorative materials. Very recent investigations published in 2025 and 2026 have continued to explore the optical behavior and clinical performance of contemporary CAD/CAM and additively manufactured restorative materials, with particular attention to their resistance to discoloration and long-term esthetic stability. These studies have reported that modern hybrid ceramic and resin-based materials demonstrate improved color stability under simulated clinical conditions, including exposure to staining agents, mouthwashes, and acidic environments [46,47,48,49]. Furthermore, recent evidence suggests that advancements in material formulation and surface finishing protocols may contribute to enhanced resistance to chromatic alteration and improved durability of esthetic restorations fabricated using digital workflows. Such findings reinforce the clinical relevance of ongoing research on the optical performance of novel restorative materials and support the continued integration of additive manufacturing technologies into routine esthetic dentistry.

From a clinical perspective, the findings of the present study suggest that 3D-printed ceramic-filled hybrid veneers may represent a viable option for the esthetic management of discolored teeth, particularly when adequate restoration thickness and material selection are considered during treatment planning.

## 5. Conclusions

Within the limitations of this in vitro pilot study, 3D-printed ceramic-filled hybrid veneers demonstrated the ability to produce substantial and clinically relevant modifications in the color of discolored dental substrates. Spectrophotometric analysis revealed significant reductions in chromatic intensity following veneer placement, indicating an effective masking of the underlying discoloration.

The color differences observed after a two-month immersion period were minimal and remained within or close to perceptibility thresholds, suggesting favorable short-term color stability of the tested material under the experimental conditions. However, the findings in this study should be interpreted with caution due to the in vitro design, the use of an artificial discoloration model, and the absence of definitive resin cementation and comparative control groups. The aging protocol was also limited and did not fully replicate the complexity of the oral environment. Only 2 months immersion is insufficient for hydrolytic degradation, and long-term color stability.

Further studies incorporating clinically relevant discoloration models, different luting agents, extended aging protocols, and comparisons with established restorative materials are required to validate the long-term optical performance and clinical applicability of 3D-printed ceramic-filled hybrid veneer restorations.

## Figures and Tables

**Table 1:** Tooth preparation parameters used for veneer fabrication.

Parameter	Value
Vestibular reduction	~0.5 mm
Incisal reduction	~1 mm
Finish line design	Chamfer
Finish line depth	~0.5 mm
Magnification	4×
Preparation burs	Diamond burs (Komet Dental)

**Table 2:** CIELAB color coordinates of the prepared teeth after creation of artificial discoloration (T0).

Sample	Shade	L*	a*	b*	C*	h*
1	2M3–A3	83.4	1.4	29.5	29.533202	87.282917
2	4M3–A3.5	77.9	5.5	48	48.314077	83.463366
3	4M3–A4	72.4	4.2	38.4	38.629005	83.758086
4	4M3–A3.5	80.9	2.9	43.1	43.197454	86.150634
5	5M3–A4	67.0	4.2	41.7	29.533202	87.282917
6	5M3–A4	67	1.7	30.2	41.910977	84.248598
7	2M3–A3	81.3	4.3	42.3	30.24781	86.778141
8	4M3–A3.5	78.5	2.8	40.6	42.517996	84.195545
9	5M3–A4	66.2	1.3	29.4	40.696437	86.054814
10	2M3–A3	83.1	5.3	46.8	29.428727	87.468162
11	4M3–A3.5	79.3	4.1	38.7	47.099151	83.538903
12	4M3–A4	75.4	3.9	37.8	38.916577	83.952463
13	4M3–A4	72.5	2.4	40.4	38.000658	84.109374
14	5M3–A4	68.4	1.8	29.1	40.471224	86.600286
15	2M3–A3	80.6	1.2	28.6	29.155617	86.460441
16	2M3–A3	81.3	5.3	30.8	28.625164	87.597391
17	4M2–A3	73.3	4.8	40.1	31.25268	80.236282
18	4M3–A4	75.9	3.9	38.6	40.38626	83.17413
19	4M3–A4	77.6	2.2	29.8	38.79652	84.230627
20	2M3–A3	80.1	5.6	32.8	29.881098	85.77777
21	5M3–A4	60.5	4.8	36.4	33.274615	80.311213
22	5M3–A4	67.3	5.2	40.2	36.71512	82.487856
23	5M3–A4	66.9	3.7	29.7	40.534923	82.629532
24	4M2–A3	73.4	4.1	32.5	29.929584	82.898728
25	3M2–A3.5	79.3	3.1	37.7	32.757595	82.809899

**Table 3:** CIELAB color coordinates of the specimens after two weeks of immersion in physiological saline solution (T1).

Sample	Shade	L*	a*	b*	C*	h*
1	2M3–A3	80.2	1	25.4	25.419677	87.745425
2	4M3–A4	72.1	4.4	39.9	40.141873	83.707095
3	4M3–A4	68.3	3.1	32.9	33.045726	84.617202
4	3M3–A3.5	77	2	37.1	37.153869	86.914266
5	4M3–C4	66.1	2.2	36.4	36.466423	86.541276
6	2M3–A3	78.1	1.3	25.4	25.433246	87.070095
7	4M3–A3.5	73.5	3.4	37.6	37.75341	84.833051
8	5M3–A4	62.7	1.9	35.7	35.750524	86.953519
9	2M3–A3	80.2	1	25.6	25.619524	87.763021
10	3M2–A2	75.9	4.4	21.4	21.847654	78.381476
11	4M3–A4	73.4	3.2	33.6	33.752037	84.559668
12	4M3–A4	68.7	2.9	32.7	32.828341	84.931982
13	5M3–A4	63.2	1.4	35.7	35.72744	87.754257
14	2M3–A3	77.1	1.2	24.2	24.229734	87.161212
15	2M3–A3	79.2	1	23.5	23.521267	87.563352
16	3M2–A3	70.1	4.4	25.7	26.073933	80.284798
17	4M3–A4	70.8	3.7	35.2	35.393926	83.999467
18	4M3–A4	71.6	2.9	33.3	33.426038	85.022835
19	2M3–A3	73.6	1.3	24.7	24.734187	86.987212
20	5M3–A4	58.3	4.6	27.6	27.980708	80.537678
21	5M2–A3.5	62.5	3.8	31.2	31.430558	83.055869
22	5M3–A4	61.6	4.3	35.4	35.660202	83.074273
23	3M2–A3	69.5	2.6	24.6	24.737017	83.966747
24	3M2–A3.5	74.8	3.2	27.8	27.983567	83.433701
25	4M3–A4	65.5	2.3	32.2	32.282038	85.914383

**Table 4:** CIELAB color coordinates recorded after placement of the 3D-printed veneers using neutral try-in paste (T2).

Sample	Shade	L*	a*	b*	C*	h*
1	2R1.5–A2	77.4	0.5	18.1	18.106905	88.417646
2	4M2–A4	67.3	2.4	24.5	24.61727	84.405203
3	4R1.5–D4	64.6	3	21.6	21.807338	82.092837
4	3R1.5–D3	71.3	1.2	18.9	18.938057	86.367049
5	3R1.5–C3	69.6	1.7	21.4	21.467417	85.458004
6	2R1.5–A2	75.3	0.8	21.3	21.315018	87.849057
7	4M2–A4	68.7	2.2	29.2	29.282759	85.691335
8	3M2–A3	59	0.6	23.4	23.407691	88.531199
9	2R1.5–A2	77.1	0.4	21.7	21.703686	88.943976
10	3M1–A2	73	3.2	19.1	19.366208	80.48904
11	4M2–A4	71.8	2.1	25	25.088045	85.198427
12	3R1.5–C3	65.5	1.1	20.2	20.229928	86.883011
13	4R1.5–D4	58.4	0.4	24.3	24.303292	89.056945
14	2R1.5–A2	74.2	0.3	20.8	20.802163	89.173676
15	2M1–A1	77.6	0.3	16.8	16.802678	88.97697
16	4M2–A4	67.5	2.9	23.8	23.97603	83.052828
17	3R1.5–C3	66.6	1.7	20.9	20.969025	85.349815
18	4M2–A4	66.6	0.9	24.4	24.416593	87.887589
19	2M1–A1	68	0.6	16.9	16.910648	87.966684
20	4R1.5–D4	56.9	2.7	22.3	22.462858	83.096446
21	4M2–A4	58.8	2.1	27.3	27.38065	85.601295
22	4M2–A4	57.4	2.7	29.7	29.822475	84.805571
23	3R1.5–C3	66.4	0.6	20.4	20.408822	88.315316
24	3M2–A3	71.1	1.5	21.4	21.452506	85.990498
25	3R1.5–C3	62.7	0.8	24.7	24.712952	88.144915

**Table 5:** CIELAB color coordinates recorded after two months of immersion following veneer placement (T3).

Sample	L*	a*	b*	C*	h*
1	74.4	0.5	17.1	17.107308	88.325162
2	69.7	2.3	26.6	26.699251	85.058145
3	65.3	2.9	23.8	23.97603	83.052828
4	67.9	2.7	20.8	20.974508	82.603923
5	67.2	1.7	20	20.07212	85.141537
6	76.9	0.4	20.1	20.10398	88.859936
7	69.2	1.8	28.5	28.556786	86.386119
8	58.3	0.4	22.7	22.703524	88.990487
9	78	0.7	20.5	20.511948	88.044319
10	74.5	2.6	18.5	18.681809	82.000017
11	71	1.5	25.2	25.244603	86.593556
12	66.3	1	20	20.024984	87.137595
13	59.1	0.2	24.1	24.10083	89.524527
14	75.8	0.2	19.4	19.401031	89.409343
15	76.8	0.6	17	17.010585	87.978635
16	68.4	3.3	22.8	23.037578	81.764381
17	65.8	2	19.7	19.801263	84.203031
18	67.9	1.3	23.9	23.93533	86.886561
19	68.6	0.8	16.8	16.819037	87.273689
20	57.5	2.1	21.7	21.801376	84.47246
21	59.3	1.7	26.7	26.754065	86.356872
22	58.6	2.2	28.3	28.385384	85.554851
23	67	1	19.7	19.725364	87.094079
24	72	1.1	20.2	20.229928	86.883011
25	63.5	0.6	24.1	24.107468	88.573844

**Table 6:** Mean ± SD values of the CIELAB color coordinates at the four experimental stages.

Stage	Mean L*	SD L*	Mean a*	SD a*	Mean b*	SD b*	Mean C*	SD C*	Mean h*	SD h*
T0	74.88	6.26	3.59	1.42	36.53	5.87	36.56	6.10	84.86	2.28
T1	70.96	6.14	2.70	1.37	30.59	5.81	30.72	5.92	85.09	2.56
T2	67.71	6.23	1.47	1.35	22.32	5.41	22.47	5.47	86.63	2.29
T3	67.96	6.36	1.42	1.37	21.93	5.52	22.05	5.58	86.57	2.40

**Table 7:** Mean color differences calculated between selected experimental stages using three color difference formulas (ΔE*ab, ΔE94, and ΔE00).

Comparison	ΔE*ab (Mean ± SD)	ΔE94 (Mean ± SD)	ΔE00 (Mean ± SD)
T0–T2	16.86 ± 4.35	14.92 ± 3.88	12.41 ± 3.21
T2–T3	1.43 ± 0.79	1.21 ± 0.66	0.98 ± 0.54

**Table 8:** Friedman test results for repeated measurements of CIELAB coordinates.

Coordinate	χ^2^	df	*p*-Value
L*	38.52	3	<0.001
a*	31.74	3	<0.001
b*	42.16	3	<0.001

**Table 9:** Pairwise comparisons using the Wilcoxon signed-rank test.

Comparison	L*	a*	b*
T0 vs. T1	*p* < 0.01	*p* < 0.05	*p* < 0.01
T1 vs. T2	*p* < 0.01	*p* < 0.01	*p* < 0.001
T2 vs. T3	n.s.	n.s.	n.s.

n.s. = not statistically significant.

## Data Availability

The data supporting the results of this study are available from the corresponding author upon reasonable request, in accordance with institutional policies and ethical regulations.

## Abbreviations

The following abbreviations are used in this manuscript:

3D	Three-dimensional
CAD/CAM	Computer-aided design/computer-aided manufacturing
SLA	Stereolithography
n.s.	not statistically significant
